# Probing the Functions of Carbohydrate Binding Modules in the CBEL Protein from the Oomycete *Phytophthora parasitica*

**DOI:** 10.1371/journal.pone.0137481

**Published:** 2015-09-21

**Authors:** Thomas Martinez, Hélène Texier, Virginie Nahoum, Claude Lafitte, Gianluca Cioci, Laurent Heux, Bernard Dumas, Michael O’Donohue, Elodie Gaulin, Claire Dumon

**Affiliations:** 1 Université Toulouse 3, UPS, Laboratoire de Recherche en Sciences Végétales, 24 chemin de Borde Rouge, BP42617, Auzeville, F-31326, Castanet-Tolosan, France; 2 CNRS, Laboratoire de Recherche en Sciences Végétales, 24 chemin de Borde Rouge, BP42617, Auzeville, F-31326, Castanet-Tolosan, France; 3 Université de Toulouse; INSA, UPS, INP, LISBP, 135 Avenue de Rangueil, F-31077 Toulouse, France; 4 CNRS, UMR5504, F-31400 Toulouse, France; 5 INRA, UMR792 Ingénierie des Systèmes Biologiques et des Procédés, F-31400 Toulouse, France; 6 Cinabio ADISSEO France SAS, Hall Gilbert Durand 3, 135 avenue de Rangueil, 31077 Toulouse, France; 7 Université de Toulouse, UPS, IPBS, Toulouse, F-31077, France; 8 Institut de Pharmacologie et de Biologie Structurale (IPBS), Centre National de la Recherche Scientifique (CNRS), Toulouse, F-31077, France; 9 CERMAV, CNRS, Grenoble, France; Ghent University, BELGIUM

## Abstract

Oomycetes are microorganisms that are distantly related to true fungi and many members of this phylum are major plant pathogens. Oomycetes express proteins that are able to interact with plant cell wall polysaccharides, such as cellulose. This interaction is thought to be mediated by carbohydrate-binding modules that are classified into CBM family 1 in the CAZy database. In this study, the two CBMs (1–1 and 1–2) that form part of the cell wall glycoprotein, CBEL, from *Phytophthora parasitica* have been submitted to detailed characterization, first to better quantify their interaction with cellulose and second to determine whether these CBMs can be useful for biotechnological applications, such as biomass hydrolysis. A variety of biophysical techniques were used to study the interaction of the CBMs with various substrates and the data obtained indicate that CBEL’s CBM1-1 exhibits much greater cellulose binding ability than CBM1-2. Engineering of the family 11 xylanase from *Talaromyces versatilis* (*Tv*XynB), an enzyme that naturally bears a fungal family 1 CBM, has produced two variants. The first one lacks its native CBM, whereas the second contains the CBEL CBM1-1. The study of these enzymes has revealed that wild type *Tv*XynB binds to cellulose, via its CBM1, and that the substitution of its CBM by oomycetal CBM1-1 does not affect its activity on wheat straw. However, intriguingly the addition of CBEL during the hydrolysis of wheat straw actually potentiates the action of *Tv*XynB variant lacking a CBM1. This suggests that the potentiating effect of CBM1-1 might not require the formation of a covalent linkage to *Tv*XynB.

## Introduction

Although plant cell walls constitute the most abundant source of renewable carbon on Earth, the industrial extraction of their constituent sugars is difficult, because the polysaccharides are chemically complex, structured and interlinked, and are embedded in a matrix of lignin, which is a high molecular weight, amorphous polyphenolic polymer [1]. To overcome these difficulties, microorganisms that degrade plant cell walls in natural ecosystems have developed a number of strategies, which include plant cell wall-specific enzymes and related proteins that together surmount the many natural obstacles that confer recalcitrance to plant cell wall [2].

Carbohydrate binding modules (CBMs) are a diverse group of protein modules that are mainly found appended to carbohydrate-acting enzymes (CAZymes). Within the Carbohydrate Active enzyme database (http://www.cazy.org/), CBMs are classified into 71 families (as of July 2015) based on amino acid sequence similarity [3]. Among the reported targets of CBMs, several have been shown to bind to crystalline cellulose, while others bind to amorphous cellulose, chitin, β-1,3-glucans, xylan, mannan, galactan or starch, with association constants ranging from 10^3^ M^–1^ to 10^6^ M^–1^ [4, 5]. In this context, the main proposed role for CBMs is to bind to polysaccharides, thus increasing effective enzyme concentration and enhancing enzyme activity [6, 7]. Furthermore, it was also demonstrated that CBMs can potentiate enzyme activity by targeting polymers other than the substrate of the catalytic domain [8]. This study suggests that the possession of a CBM would confer an advantage for polysaccharide degrading enzymes in the context of complex substrate (*e*.*g*. lignocellulosic biomass).

The family 1 carbohydrate-binding module (CBM1) is mostly found in fungal cellulose-degrading enzymes [9] where they are thought to efficiently direct cellulose binding to crystalline cellulose structure [10]. Several studies have shown that CBM1 alone disrupts the crystalline structure of cellulose and facilitates hydrolysis by rendering the substrate more accessible [11–13]. Accordingly, the removal of a CBM1 from fungal cellulases frequently leads to reduced ligand affinity and to diminished hydrolytic activity on insoluble substrates [7, 14–16]. CBM1s are also detected in fungal xylanases (*i*.*e* GH10 and GH11), either at the N- or C-terminus [9]. The presence of CBM1s in fungal xylanases does not always appear to be beneficial for the degradation of soluble and insoluble xylans [17]. However, the exact role of this module in the hydrolysis of lignocellulosic substrates is still poorly understood and requires further investigation.

Structural studies have revealed that all known CBMs display one of three binding site topologies (type A, B or C) [5]. Regarding type A CBMs, which are all small domains (32 to 36 aa long), structural analysis of the family 1 CBMs belonging to the cellobiohydrolase I from *Trichoderma reesei* has revealed that these binding domains display a wedge-shaped structure that is characterized by a flat hydrophobic surface composed of three aromatic residues (Y5, Y31 and Y32) that, along with polar residues (Q7 and N29), form the polysaccharide binding interface [18]. Moreover, CBM1s contain four cysteine residues forming two disulfide bridges that are required for the correct folding of the domain [18]. Overall, it is thought that these two structural features are common to all fungal CBM1s [9].

Although most CBM1s are of fungal origin, some are produced by oomycetes [19], fungal-like, filamentous microorganisms that are actually more closely related to the heterokont algae (i.e., diatoms, brown algae) than to true Fungi. The Oomycota phylum contains more than 800 species that occupy terrestrial or aquatic environments and includes some of the most devastating plant pathogens (e.g. the causal agent of potato blight *Phytophthora infestans* or the sudden oak death pathogen *Phytophthora ramorum*) [20]. Unlike the cell-wall degrading enzymes from true Fungi, many of those isolated from oomycetal species do not possess CBM1s [19, 21]. On the other hand, in oomycetal species CBM1s have been detected as single domains, or as part of multidomain proteins [21, 22]. Regarding the latter, these are often proteins that are composed of CBM1s that are appended to a so-called PAN/Apple module, a protein domain that can fulfil diverse biological functions by mediating protein-protein or protein-carbohydrate interactions [23–25]. Significantly, although CBM1s of oomycetal origin display some specific features, they also share conserved cysteines and aromatic residues with the CBM1s of fungal origin [19].

The **c**ellulose-**b**inding **e**licitor **l**ectin (**CBEL**) from *Phytophthora parasitica* represents the best described oomycetal CBM1-containing protein. This non-enzymatic cell wall glycoprotein harbors two homologous, but non-identical copies of CBM1-PAN/Apple association, with the two repeats being separated by a threonine/proline-rich linker region [26]. This glycoprotein is described as an elicitor due to its capacity to trigger plant immune responses [27]. *In vitro*, CBEL has been shown to bind to tobacco cell walls and crystalline cellulose (i.e. Avicel) [27]. Previous findings show that CBEL is a lectin that acts as a structural protein in the cellulosic cell wall of *Phytophthora* and mediates the attachment of the microorganism to host surfaces [28]. Structural prediction using *in silico* modelling has shown that oomycetal CBM1s harbour five cysteines, four of these being engaged in two disulphide bridges, and has suggested that amino acids F25, Y51 and Y52 in CBM1-1 and Y162, F187 and Y188 in CBM1-2 are surface-exposed and confer cellulose binding ability to CBEL [19]. Furthermore, experimental work has revealed that the affinity for cellulose and the elicitor activity of CBEL are both significantly diminished when Y52 and Y188 (located in CBM1-1 and CBM1-2 respectively of the glycoprotein) were mutated to alanine [27]. However, apart from this study it is clear that so far CBM1s from oomycetes have been much less characterised than their fungal counterparts.

In this study, we set out to better characterize CBEL and in particular it’s CBM1s. The motivation underlying this study was a desire to better understand the polysaccharide binding capabilities of the oomycetal CBM1s and to determine whether these could be useful to enhance the degradation of lignocellulosic biomass. To reach these aims, the CBEL-associated CBM1s were characterized using a variety of techniques and were also linked to a fungal xylanase using a protein engineering strategy. Overall, the data described in this work confirm the polysaccharide binding ability of the oomycetal CBM1s and reveal the potential of these protein modules for the development of biomass degradation strategies.

## Materials and Methods

### General material and chemicals

Unless otherwise stated, all chemicals were of analytical grade and purchased from Sigma–Aldrich (St. Louis, MO, USA). Restriction enzymes, *Taq* DNA polymerase and their corresponding buffers were obtained from New England Biolabs (Ipswich, MA, USA). Oligonucleotides were synthesized by Eurogentec (Angers, France). Avicel PH-101 (Ref. 11365) and birchwood xylan (BWX) (Ref. X0502) were purchased from Sigma Aldrich. Cellohexaose, sugar beet arabinan, barley mixed-linkage glucan and wheat arabinoxylan were purchased from Megazyme (Wicklow, Ireland). Wheat straw (Apache variety), harvested in 2007 in southern France, was obtained from ARD (Pomacle, France) and milled to 0.5 mm as previously described [29] and cellulose nanocrystals from cotton linters (in a 2% w/w aqueous suspension) were kindly prepared by Laurent Heux (CERMAV, Grenoble, France) using an established method [30]. The resulting nanoparticles displayed lath form (200 ± 60 nm, length, 20 ± 10 nm, width and 7 nm height).

### Heterologous expression of proteins in *E*. *coli*

#### Cloning of *Tv*XynB in *E*.*coli*

XynB from *Talaromyces versatilis* (GenBank AJ489605.1) was cloned into pET28a between NdeI and HindIII (generating pET-*Tv*XynB), using PCR to amplify the two exons of the *Xyn*B gene, exon1 primers E1fo 5’-ATACTTCATATGGCTGAGGCGATCAACTACAACC-3’ and E1re 5’-CTGTAGGTGATGGGTTAGCATCACCTGGTTGCCAACC-3’, and exon 2 primers were E2fo 5’-GGCAACCAGGTGATGCTAACCCCATCACCTACAGCGGC-3’ and E2re 5’-ATTCAAAGCTTCTTGGCACTGGCTGTAGTAAGCG-3’. Using the previously prepared construction as template DNA, *Tv*XynBΔCBM was obtained by PCR, amplifying only the xylanase-encoding sequence (nucleotides 1 to 693) of the previously cloned *Xyn*B sequence using primers E1fo and E2SSCBM: 5’-ATTCAAAGCTTCAGCAGCACCGGTGCCTCC-3’ and cloned into pET28 (generating pET-*Tv*XynBΔCBM). The sequence encoding the first copy of CBM1 (designated CBM1-1) from the *Phytophthora parasitica* CBEL (amino acids 23 to 55) was amplified by PCR using the CBEL cDNA [27] as the template. The amplicon was cloned into pET28a- *Tv*XynBΔCBM using *Hind*III and *Xho*I, thus creating a new cassette encoding the fusion protein *Tv*XynB-CBM1-1.

#### Protein expression in *E*. *coli*

CBEL from *Phytophthora parasitica* (GenBank ID: X97205) and variants thereof (CBEL_Y52A_Y188A_, CBEL_Y52A_, CBEL_Y188A_) were expressed in *E*. *coli* strain BL21 DE3 as previously described [27]. Briefly, protein expression was induced by adding isopropylthio-β-D-1-thiogalactopyranoside (500 μM final) to a bacterial culture (incubated at 37°C with shaking) when an absorbance (600nm) reading of 0.5 (approximately 3 h after inoculation) was achieved. The culture was pursued for a further 4 h at 37°C and then the cells were recovered by centrifugation (5000 g, 20 min, 4°C) and lyzed to extract protein inclusion bodies. Urea-solubilized inclusion bodies were dialyzed at 4°C against 10 L of sodium acetate buffer (100 mM, pH 5.2) and applied to a CM-Sepharose chromatography column equilibrated in the same buffer. Proteins were separated by applying a NaCl gradient (0 to 1 M) and eluted proteins were combined and neutralized by exhaustive dialysis (100 volumes) at 4°C for 48 h against water. The proteins *Tv*XynB, *Tv*XynBΔCBM and *Tv*XynB-CBM1-1 were expressed in *E*. *coli* strain Tuner DE3 following a similar procedure, except that expression was induced using a lower concentration of IPTG (200 μM final) and growth was pursued for a further 16 h at 16°C after induction. After recovery of a cell pellet by centrifugation (5000 g, 20 min, 4°C) and cell lysis by sonication (10 min, using pulses of 0.5 s), the cell supernatant containing the soluble protein fraction was applied to a cobalt affinity chromatography column (Talon) in 20 mM Tris-Hcl pH 8.0 300 mM NaCl buffer (Talon buffer). His-tagged proteins were eluted using a gradient of imidazole (0 to 200 mM) and then all fractions containing the recombinant protein were combined and dialyzed against 100 volumes of 20mM Tris-HCl, pH 7.0 buffer at 4°C for 24 h. The purified enzymes were adjudged homogeneous after examination of a SDS-PAGE and stored at 4°C. Protein concentrations were determined by measuring absorbance at 280 nm and applying the Lambert–Beer equation. Theoretical molar extinction coefficients were calculated using ProtParam online software [31].

#### Solid state depletion assays

Adsorption assays to Avicel cellulose were performed in 0.1 mM sodium acetate buffer, pH 5. Proteins at concentrations in the range 0–25 μM were incubated under continuous agitation (1100 rpm, thermomixer Biorad) for 16 h at 25°C with 2.5% Avicel, before recovery of a supernatant and a solid fraction using centrifugation (10 min, 10, 000 g, 22°C). Upon removal of the supernatant, the solid fraction was once again submitted to centrifugation in order to ensure that the entire liquid fraction was recovered and then the concentration of the unbound, soluble protein fraction (i.e. in the supernatant) was determined using the Bradford method (Protein Reagent Assay, Bio-Rad). The bound protein concentration was then deduced by subtracting the unbound concentration from the initial protein concentration. The binding parameters (*K*_D_ and B_max_) were calculated by fitting the data to a single-site Langmuir isotherm using Sigma-plot software (version 12.0). Each experiment was performed in triplicate.

#### Fluorescence spectroscopy experiments

Fluorescence spectra were acquired at 25°C using a Cary Eclipse Agilent technology spectrofluorimeter and 5 mm × 5 mm quartz cuvettes. The excitation and emission wavelengths were 295 and 300–500 (scan) nm respectively and slit widths were 5 nm. To perform the experiments, a buffered protein solution 7 μM protein (final concentration) in 50 mM HEPES, pH 7.5 was placed in a cuvette with 0, 0.05, 0.1, 0.5, 1 and 1.5% w/v of final concentration of a suspension of cellulose nanocrystals. Spectra were recorded at 2 nm intervals until no further spectral changes were detected. At this point it was assumed that binding was complete. All titrations were performed in triplicate. When a wavelength shift of intensity E_max_ was observed, the barycentric mean wavelength of the integral between 300 to 500 nm was calculated as previously described [32]. The dissociation constant *K*_D_ and B_max_ were calculated from the curve by fitting the data to a one site binding (hyperbola) model contained in the Sigma-plot software suite (version 12.0).

#### Isothermal titration calorimetry (ITC)

Isothermal titration calorimetry (ITC) experiments were conducted at 25°C in 50 mM HEPES, pH 7.4 using a Microcal ITC200 instrument (GE Healthcare, Little Chalfont, UK). To ensure minimal buffer mismatch, prior to the experiment the proteins were dialyzed against the buffer and the ligand molecules were directly solubilized in it. Experiments consisted of a series of 20 x 2 μl injections of cellohexaose (1.6 to 3 mM) into a protein solution (CBEL or CBEL_Y52A_Y188A_ at 80 to 110 μM) contained in the thermostatic cell (initial delay of 60 s, duration of 4 s and spacing of 100 s). ITC experiments were systematically performed in duplicate. A titration of the ligand in the sample cell containing only buffer was subtracted from the actual binding experiment before data analysis. The corrected binding isotherms were fitted to a two sequential binding sites model using non-linear least squares analysis to obtain the value of the equilibrium binding constant (*K*_A_), and enthalpy changes (ΔH) associated with ligand binding.

#### Measurement of xylanase activity

All enzyme activity measurements were performed in triplicate by monitoring the release of reducing sugars, which were quantified using the standard DNS method. During the reaction aliquots (100 μL) were removed at regular intervals and immediately mixed with an equal volume of 3,5-dinitrosalicylic acid (DNS) reagent [33]. Afterwards, the absorbance (540 nm) of each sample was measured and compared to a standard curve prepared using a xylose solution at different concentrations in order to determine concentration of reducing xylose equivalents. No correction was made for the fact that the actual product of hydrolysis is mainly xylo-oligosacharides.

#### Activity of *Tv*XynB and derivatives on birchwood xylan

First, to determine the optimal operating conditions for recombinant *Tv*XynB, enzyme activity was measured on 5 g/l birchwood xylan (BWX) at 40°C using 50 mM citrate-phosphate buffers of pH ranging from 2.5 to 5. Once the optimal pH for activity was determined, the reaction temperature was varied, while operating at the optimal pH (3.0). To measure activities, the DNS method was employed and reactions were performed as described in the xylanase activity section unless stated otherwise. Activities were expressed in units, where 1 unit is defined as the quantity of enzyme required to release 1 μmole of reducing xylose equivalent per minute. Once the optimal conditions for *Tv*XynB were determined, enzyme kinetics were studied by performing reactions in 50 mM citrate-phosphate buffer, pH 3.0 at 40°C using eight different concentrations of BWX in the range 0.5–25 g. l^−1^. Reactions were monitored using the DNS and initial velocities were plotted as a function of substrate concentration using SigmaPlot Ver12.0 enzyme kinetics package. Data were processed using the non-linear regression algorithm and values of *k*_cat_ and apparent *K*_m_ were obtained. Thermostability was monitored by preincubating the enzymes and variants (100 mM) at 40°C, 45°C, 50°C and 55°C for up to 24 h in 50 mM citrate-phosphate buffer, pH 3.0. Residual xylanase activity in each case was then assayed as described above all experiments were performed in triplicate.

#### Determination of activity on wheat straw

Xylanase activity on milled wheat straw (WS) was determined as described above (at 40°C in 50 mM citrate-phosphate buffer, pH 3.0) using 2% (w/v) WS and XynB, XynBΔCBM or XynB-CBM1-1 at final protein concentrations of 0, 0.1, 0.25 0.5, 1 and 2.5 mg of protein per g of substrate. After 24-h incubation under continuous agitation, the reaction mixture was centrifuged to remove solid matter (10 min, 10,000 x *g*, RT) and the amount of reducing sugars released into the supernatant was quantified by DNS method., Experiments were performed in triplicate and student’s t-test was realized using p-value < 0.05, and *t*-test compared to *Tv*XynB, the wild type reference. Enzyme activity was expressed in units as described above.

#### Monitoring synergy between CBEL and *Tv*XynB variants

In order to detect any synergistic effects, procured by combining CBEL with *Tv*XynB or one of its derivatives at 0.1 mg/g of WS, activities were measured using WS substrate as described above, except that CBEL was added to a final concentration of 10 mg/g of WS and reaction were performed in 2 mL eppendorf tubes at 40°C for 24 h with continuous agitation (using a Biorad Thermomixer operating at 1,400 rpm). Control experiments that only contained CBEL were also performed. For analysis, the reaction mixture was centrifuged (10,000 x *g* for 10 min) and the amount of reducing sugars in the supernatant was quantified using the DNS assay. Experiments were performed in triplicate, and student’s t-test was realized using p-value < 0.05, and *t*-test compared compared to *Tv*XynBΔCBM reference. Activities were expressed in units as described above.

## Results and Discussion

**Fig 1:**
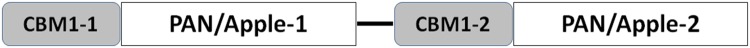
Schematic representation of CBEL protein domains. CBM1s and PAN/Apple are numbered from the N- to the C-terminus of the protein. CBM1s and PAN/Apple domains are symbolized by grey and white boxes respectively. The linker is represented by a black line.

CBEL is a glycoprotein from *Phytophthora parasitica* that is composed of two CBM1s and two non-catalytic modules designated PAN/APPLE and are known to bind to crystalline cellulose *in vitro* (Fig 1). A previous mutagenesis study has provided evidence that Y52 and Y188 located on the surface of CBM1-1 and CBM1-2 respectively are important for cellulose binding, since the double mutant CBEL_Y52-Y188_ was deprived of binding ability [27]. Since the binding properties of CBEL were studied exclusively with regard to elicitor activity, we decided to better characterize and quantify the binding properties of CBEL, using the double mutant CBEL_Y52-Y188_ and the single mutants: CBEL_Y52A_ and CBEL_Y188A._

### CBEL-crystalline cellulose binding capacity

#### Solid state depletion assay

A solid state depletion assay using Avicel was performed in order to quantify CBEL binding to this crystalline cellulose. In this assay, wild type CBEL displayed ability to bind the ligand, while use of the double mutant, CBEL_Y52A_Y188A,_ failed to reveal any detectable binding, consistent with the previous finding that the aromatic residues confer binding ability to CBEL (Table 1). Similarly, a variant of CBEL bearing a single mutation in its CBM1-1 (CBEL_Y52A_) also displayed 15-fold decreased binding to Avicel. This is in sharp contrast to the binding ability displayed by the other single mutant CBEL_Y188A_ (mutated in CBM1-2), which was similar to that of wild type CBEL (Table 1). Interestingly, this latter observation suggests that CBM1-1 is the main determinant of cellulose binding in CBEL and that Y52 plays a key in this function.

**Table 1:** Characterization of CBEL and *Tv*XynB binding to Avicel by solid state depletion assay.

Protein	B_max_	*K*_D_
	(μmol.g^-1^) ^1^	(μmol.L^-1^) ^2^
CBEL_WT_	0.094 ± 0.004	4.3 ± 0.4
CBEL_Y188A_	0.125 ± 0.003	6.3 ±0.5
CBEL_Y52A_	0.29 ± 0.07	65.3 ± 20.6
CBEL_Y52A_Y188A_	N.D.	N.D.
*Tv*XynB	0.42 ± 0.02	0.6 ± 0.1
*Tv*XynB-CBM1-1	0.092 ± 0.002	2.1 ± 0.2
*Tv*XynBΔCBM	N.D.	N.D.

^1^ Bmax is expressed in μmol of protein per g of substrate.

^2^ *K*_D_ is expressed as μmol.L^-1^ of proteins.

#### Fluorescence spectroscopic analysis of CBM-ligand interactions

To further investigate the binding properties of the CBEL variants, the interaction of these with cellulose nanocrystals was studied using fluorescence spectrophotometry. The advantage of using cellulose nanocrystals was that these are water soluble and mimic the cellulose surface [34, 35]. In these experiments, the calculated value for *K*_D_ was 2.5 g.L^-1^ for CBEL, but binding was not detected in the case of CBEL_Y52A_Y188A_, a result that is once again consistent with the other data reported in this study (Table 2). Likewise, as before the binding ability of the single mutant CBEL_Y52A_ was found to be severely reduced with the *K*_D_ value being increased approximately 40-fold, while that of CBEL_Y188A_ was only increased 6-fold (Table 2). Together, these data support the view that CBEL binding to cellulosic compounds is mainly driven by CBM1-1 and that the binding ability of CBM1-2 is much lower. In this respect it is noteworthy that the binding ability of CBM1-1 to Avicel is comparable to that of fungal CBM1s and that residue Y52 in CBM1-1 appears to be a functional homolog of Y32 in the CBM1 of *T*. *reesei* cellobiohydrolase I, the latter being known to be a cellulose binding determinant [36, 37]. However, the results also raise a new question concerning the possible specificity and thus the biological role of CBM1-2.

**Table 2:** Characterization of CBEL binding to cellulose nanocrystals by fluorescence spectroscopy.

Protein	*K*_D_ (% w/v)
CBEL_WT_	0.24 ± 0.07
CBEL_Y188A_	1.6 ± 0.3
CBEL_Y52A_	10.4 ± 3.7
CBEL_Y52A_Y188A_	N.D

N.D: no binding detected.

### CBEL-cellooligosaccharide binding capacity

**Fig 2:**
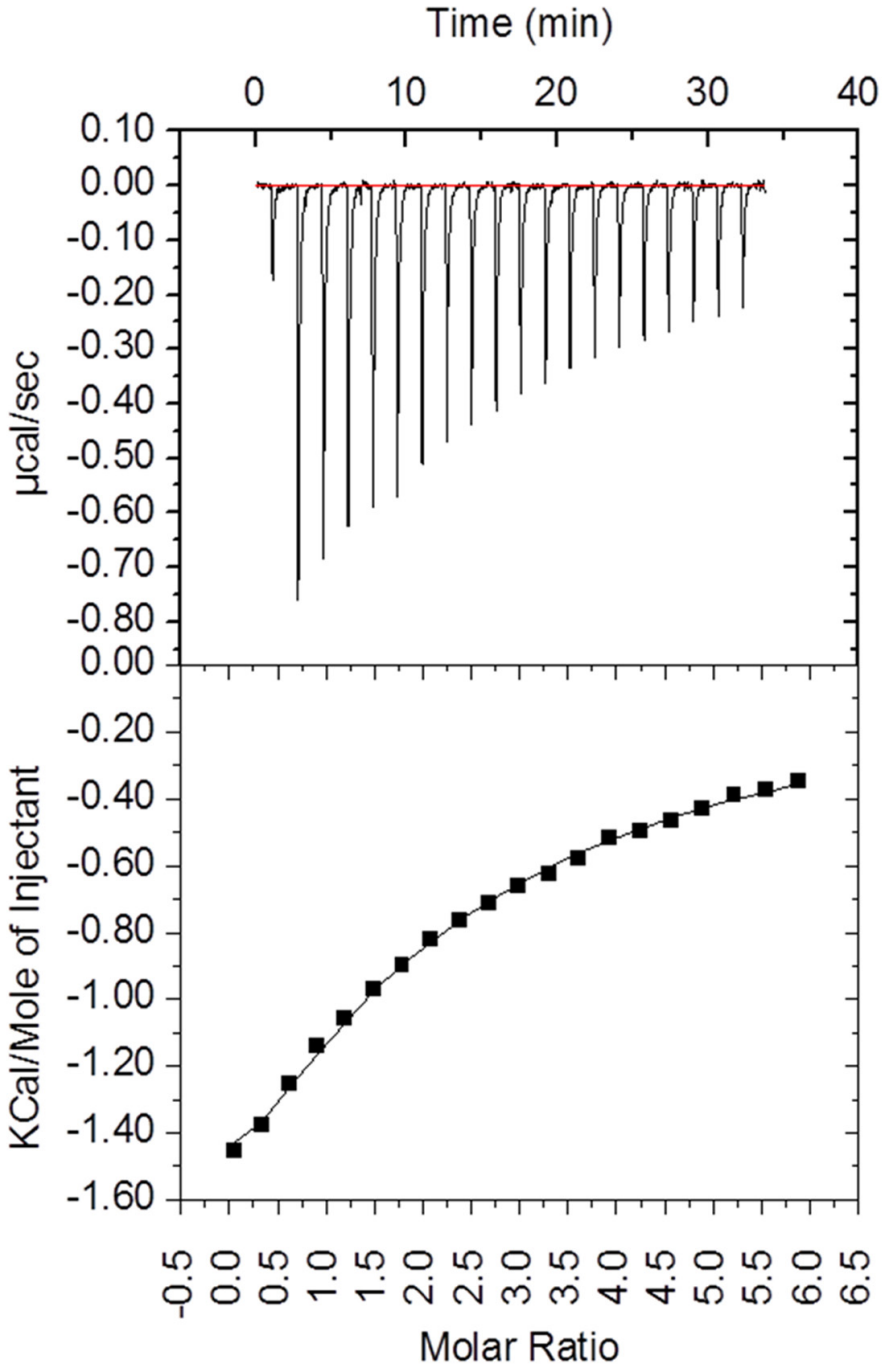
Binding interaction between CBEL and cellohexaose as investigated by isothermal titration calorimetry (ITC). The upper panel shows the thermogram representing the heat of binding. The lower panel shows the titration curve. Fitting procedure was performed using a two sequential binding site model.

In order to obtain detailed information on binding abilities of CBEL, isothermal titration calorimetry (ITC) experiments (Fig 2) were done in the presence of cellohexaose, the longest soluble cello-oligosaccharide commercially available. Upon analysis of the ITC data, and based on the previous results it appeared that the CBEL binding to cellohexaose titration could be best analysed using a two sequential binding sites model, with higher (*K*_D_1 = 52 μM, ΔH1 = -1967 kcal mol^-1^) and lower affinity (*K*_D_2 ~ 800 μM, ΔH1 = -9156 kcal mol^-1^) binding sites (Fig 2). Although the affinity observed is weak, it is consistent with the affinity observed for cellulases with cellohexaose and with other CBM families [4, 38]. In the case of CBEL_Y52-Y188_, under the experimental conditions, we were unable to observe any binding to cellohexaose (data not shown), which supports the hypothesis that the residues Y52 and Y188 are key determinants of CBEL’s sugar binding ability.

### The action of CBEL on wheat straw

Some fungal CBM1s display the ability to disrupt the surface of cellulose via a non-catalytic mechanism [11–13]. Therefore, to investigate whether CBEL could exhibit the ability to alter cellulose structure in a similar manner, recombinant CBEL was incubated with wheat straw. CBEL induced the release of a small, but significant amount of reducing sugars (905 μM), compared to the BSA control (103 μM). Although the activity of PAN/Apple domains on the substrate could not be excluded in this assay, this result supports the view that CBM1s from CBEL somehow modify cell wall polysaccharides. However, we were unable to pursue and complete this study, because it proved impossible to produce CBM1-1 on its own in *E*. *coli* in order to evaluate its impact on wheat straw. Nevertheless, it was previously shown using a partially folded CBM1-1 synthetic peptide, that it triggers plant immune responses [27, 39]. To further pursue our investigation of action of CBEL on wheat straw, we also combined CBEL with a commercial cellulolytic cocktail. However, in this case the action of the cocktail was not accentuated, implying that the cocktail alone was able to access the polysaccharides that were targeted by CBEL in the previous experiment (data not shown).

### Appendage of CBM1-1 onto a fungal endoxylanase

Addition of xylanases to cellulose mixture has been reported to enhance glucose release by increasing the accessibility of the cellulose to cellulases through the removal of hemicellulose [40, 41]. Moreover the precise role of CBM1s appended to fungal xylanases in the hydrolysis of lignocellulose is unclear, even if it is known that CBMs potentiate enzyme action on plant cell walls by targeting polymers that are in close vicinity to the partner enzyme’s substrate [8]. In this context we investigated whether oomycetal CBM1s can potentiate the action of a fungal xylanase on wheat straw. Therefore a, chimeric enzyme, *Tv*XynB-CBM1-1, was created by replacing the CBM1 of the *Talaromyces versatilis* xylanase B, *Tv*XynB [42] with CBEL CBM1-1. Likewise, for the completeness of the study, a truncated mutant was created, *Tv*XynBΔCBM, which lacks a CBM1. Both proteins were successfully expressed in a soluble form in *E*. *coli* and could be purified to near homogeneity. *Tv*XynB and *Tv*XynBΔCBM recombinant proteins displayed apparent molecular weights of 27.8 and 26 kDa, which are consistent with the expected molecular weight.

#### Properties of *Tv*XynB and its variants

Biochemical characterization of *Tv*XynB and the variants revealed that the three enzymes displayed very similar properties, which were actually rather consistent with the properties of the *Tv*XynB expressed in *Pichia pastoris*, [26]. Importantly, all of the enzymes were optimally active in the pH range 3.0–3.5 with a maximum activity at pH 3.0, which is relatively low, but not unusual for fungal endoxylanases [43], and at 55°C. However, at 55°C the enzymes described in this study were less stable (loss of 25% activity after 30 minutes) than *Tv*XynB expressed in *P*. *pastoris* [26]. Therefore, experiments were actually performed at 40°C, thus ensuring that the enzymes would remain stable for at least 24h (data not shown). This difference in thermostability of recombinant *Tv*XynB expressed in *E*. *coli* and *P*. *pastoris* might be due to glycosylation, which was proven to be extensive in *P*. *pastoris*, but totally absent in *E*. *coli*. Regarding specific activity, all three enzymes *Tv*XynB, *Tv*XynBΔCBM and *Tv*XynB-CBM1-1 exhibited similar behaviour (1515 ± 162 IU. mg^-1^ protein) on birchwood xylan (BWX) (Table 3). Similarly, kinetic analysis revealed almost identical values for *k*_cat_ and apparent *K*_m_ (Table 3), confirming that neither the presence of the wild type CBM1 nor that of the CBEL-derived domain modulated the activity of the catalytic domain on BWX. This result is consistent with previous findings that indicate that CBM1s generally target cellulose, even when they are appended to catalytic modules that do not hydrolyze cellulose [38, 44]. This conclusion was supported by depletion assays, which revealed that both the wild type *Tv*XynB and the variant *Tv*XynB-CBM1-1 were able to bind to Avicel, whereas the *Tv*XynBΔCBM did not (Table 1). Nevertheless, binding to Avicel was not identical, since the wild type *Tv*XynB bound more strongly than *Tv*XynB-CBM1-1. Moreover, gel retardation assays using the wild type enzyme and *Tv*XynB-CBM1-1 failed to reveal interactions of any of the proteins with sugar beet arabinan, wheat arabinoxylan or mixed-linkage β-(1, 3) (1, 4)-d-glucan (data not shown).

**Table 3:** Optimal reaction conditions and kinetic parameters of *Tv*XynB and mutants on BWX.

	pH—Temp.	K_m_	*k*_cat_	*k*_cat_/K_M_	Activity
		(g/L^-1^)	(s^-1^)	(s^-1^.g.L^-1^)	(UI/mg)
*Tv*XynB	3–55	14.7 ± 3.0	734.2 ± 75.6	50.01	1516 ± 162
*Tv*XynB**Δ**CBM	3–55	14.3 ± 2.5	728.3 ± 62.3	51.07	1814 ± 155
*Tv*XynB-CBM1-1	3–55	15.0 ± 3.6	786.2 ± 99.6	52.34	1690 ± 214

#### Wheat straw hydrolysis by *Tv*XynB and its variants, and the influence of CBEL

**Fig 3:**
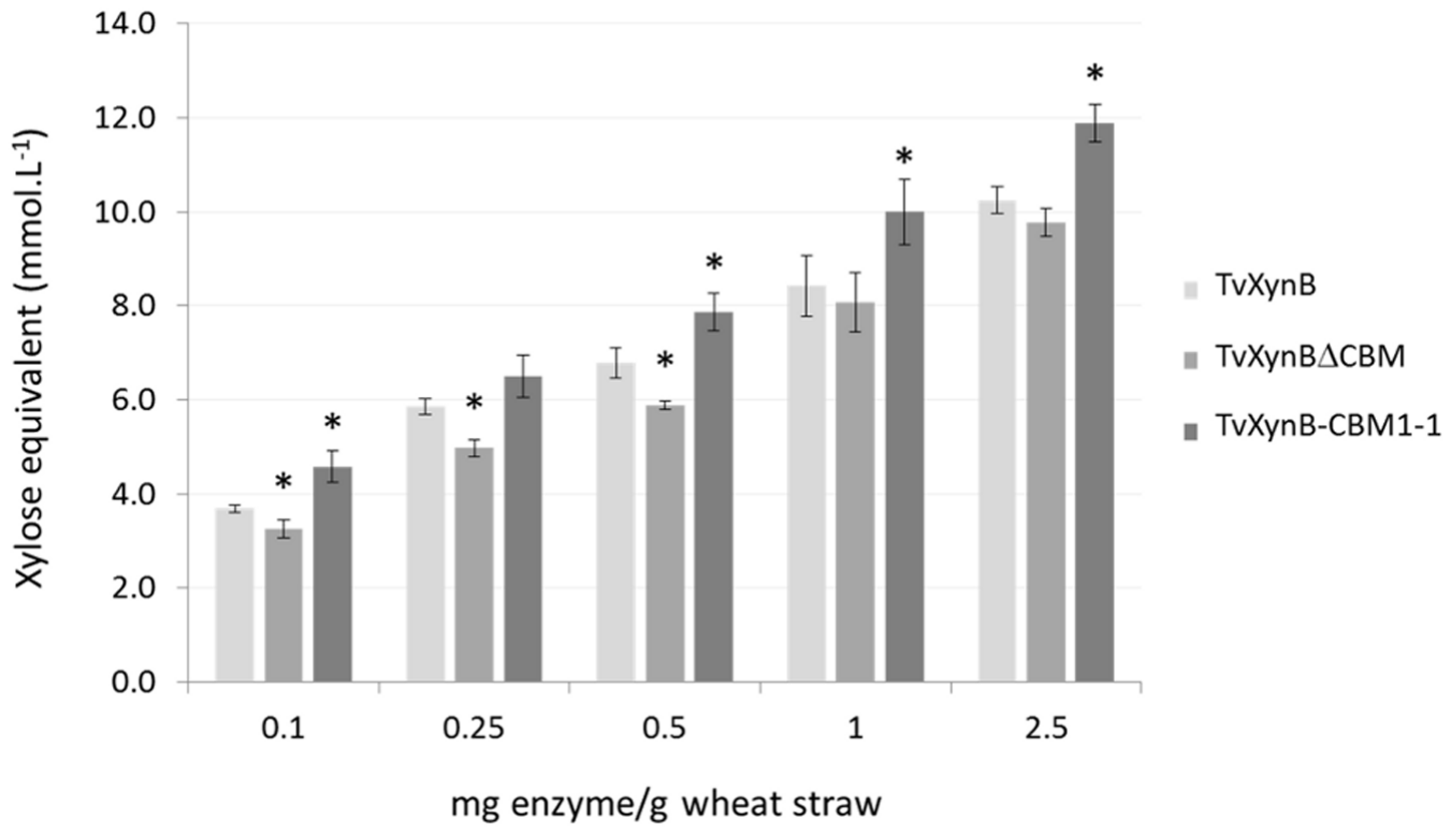
Wheat straw hydrolysis by *Tv*XynB xylanase and its derivatives. Black, grey and white bars represent *Tv*XynB-CBM1-1, *Tv*XynB and *Tv*XynBΔCBM respectively. Increasing amounts of *Tv*XynB, *Tv*XynBΔCBM and *Tv*XynB-CBM1-1 (0.1 to 0.5 mg enzyme/g substrate) were incubated with 0.02 g of wheat straw. The amount of solubilized reducing extremities was measured after 24h of hydrolysis at 40°C, pH 3.0 using the DNS assay. A control without enzyme was included and all experiments were conducted in triplicate. Asterisks indicate significant differences (p-value<0.05, *t*-test compared to *Tv*XynB, the wild type reference).

Using wheat straw as the substrate, hydrolytic properties of the *Tv*XynB-CBM1_-_1 were further investigated, comparing performance with that of the wild type enzyme and *Tv*XynBΔCBM respectively. After 24-h hydrolysis, the amount of reducing sugars released by *Tv*XynBCBM1-1 was generally higher than that observed with *Tv*XynB. At low enzyme loadings this increase in activity was roughly 10% but was more significant (16, 18 and 16%) at higher (0.5, 1 and 2.5 mg/g) loadings respectively (Fig 3). Therefore, this confirms that CBM1-1 can at least restore wild type activity in XynΔCBM and at best can enhance activity beyond wild type levels. Presumably this effect can be attributed to the binding of CBM1-1 to cellulose, which is in intimate contact with cell wall arabinoxylans.

**Fig 4:**
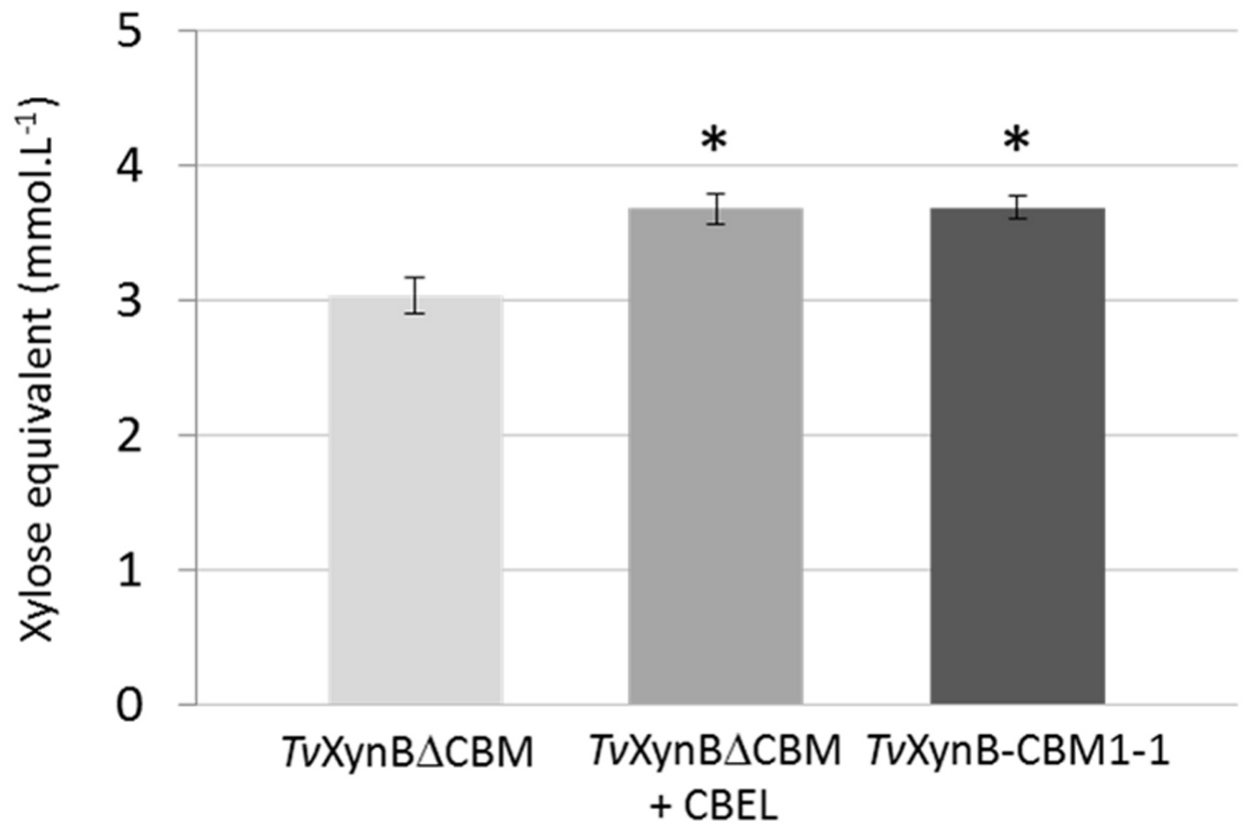
Complementation of *Tv*XynB with CBEL in Wheat straw hydrolysis. *Tv*XynBΔCBM (0.1 mg enzyme/g substrate) was incubated with or without CBEL (10 mg.g^-1^ of wheat straw) at pH 3.0 and 40°C under agitation (1400 rpm) during 24h. *Tv*XynB-CBM1-1 without CBEL is shown as control. Experiments were conducted in triplicate. Asterisks indicate significant differences (p-value<0.05, *t*-test compared to *Tv*XynBΔCBM reference).

To complete this study, wheat straw hydrolysis was also performed using a combination of either *Tv*XynBΔCBM, *Tv*XynBCBM1-1, or *Tv*XynB, and CBEL (Fig 4). This revealed that the reaction procured a yield of soluble reducing sugars that was similar to the one involving *Tv*XynB-CBM1-1. If one assumes that the PAN/Apple domains play no role here, this observation is both intriguing and confusing, since it might imply that potentiation of the activity of the xylanase module by the CBM1-1 does not require a covalent linkage. Alternatively, the binding of CBEL (via its CBM1-1) to cellulose might provoke amorphogenesis that in turn leads to the non-specific solubilisation of components that are not covalently bound in the cell wall. To pursue this investigation, and better understand the underlying phenomena, it will be necessary to renew efforts to obtain an isolated recombinant form of CBM1-1.

## Conclusions

This study provides new insight into the cellulose binding properties of CBM1s from the oomycetal protein CBEL and reveals that CBM1-1 is probably the major determinant of CBEL’s interaction with cellulose. Moreover, this work has demonstrated that the CBM1-1 from *Tv*XynB somehow potentiates the action of the xylanase, although exactly how this is achieved remains unclear. Indeed, although appending the CBM1-1 to the catalytic module of *Tv*XynB enhanced activity compared to the equivalent enzyme lacking a CBM, a similar result could be obtained by combining *Tv*XynBΔCBM with free CBEL. Therefore, although this study has shown that CBM1-1 could be interesting for the creation of chimeric enzymes, it also tantalizingly raises a number of new questions that require further investigation.
